# Progress in Psychiatry

**Published:** 1902-04-05

**Authors:** 


### Progress in Psychiatry.

**Treatment of Melancholia.**—In the treatment of melancholia a number of bodily and cerebral conditions have to be taken into account. Many of these are capable of improvement, and in proportion to the accumulated improvements which may be attained in these several respects the patient's condition as a whole may be regarded as benefited. Such bodily and cerebral conditions include constipation, cold dry skin, high arterial blood pressure with contracted peripheral arterioles, cerebral apathy as regards conduct, insomnia and cephalagia, acute mental pain (cerebral overaction as regards obsessions and reactions of fear and pain), general wasting of the body, and impulses to suicidal and dangerous acts. These conditions may be present in varying degrees of development when the patient comes under treatment, and in some patients they may be more amenable while in others they are less amenable to treatment. While these individual items have to be borne in mind a more general knowledge of the patient as regards personal and family history, social position, occupation, and heredity will help towards modification of the various lines of treatment in this or that respect. The lines of treatment as regards the conditions enunciated will now be considered *seriatim*: (*a*) Constipation. This should be treated with mercurials, *e.g.*, blue pill at night followed by black draught in the morning. Enemata may also be given. A dose of salol or beta-naphthol may also be given daily to promote intestinal disinfection. A dilated stomach should be treated by lavage. (*b*) Cold dry skin. To promote the action of the skin a mild diaphoretic mixture of ammonium acetate and sodium nitrite may be given. The latter drug also helps to produce arterial relaxation and lower blood pressure, and moreover, by their combined action diuresis is also promoted. (*c*) The remarks made in the last sentence also apply to the arterial condition in melancholia. The addition of a small quantity of sodium or potassium iodide to the nitrite will increase the effect desired. (*d*) The condition of apathy or cerebral inertia should be treated by nerve-tonics, and the exhausted cerebral centres should be recuperated by rest and sleep. Among the nerve-tonics to be administered are strychnine or nux vomica, iron, and the hypophosphites as in the Syrup Hypohosph. Co., malt extracts with or without hæmoglobin, and cod liver oil. (*e*) Insomnia. Several of the measures recommended above also tend, directly or indirectly, to abolish insomnia. Among the special hypnotics and sedatives to promote sleep in melancholia are opium, sulphonal, paraldehyde, and chloral combined with cannabis indica. The prolonged warm bath or the hot wet-pack are sedative and help to procure sleep. Massage and shampooing of the scalp before bed-time also help to promote sleep. The stomach, when secretion and digestion are defective, should not be overloaded with food, and while a due supply of carbohydrates is maintained in the food there should be restriction as regards meat, beef-tea, or meat extracts which, owing to the presence of nitrogenous extractives [1] (xanthin, creatin, and substances allied to uric acid) would tend to promote high arterial tension, cephalalgia and other untoward symptoms. (*f*) Rest in bed. This is necessary in all cases during the acute stages of the disease [2] The room should be quiet, well-ventilated, and warm. Patients resting long in bed should be kept scrupulously clean and dry, and strict attention should be paid to the evacuation of the bowels and bladder daily. Any bruises or injuries on the body should be kept clean and aseptic with the aid of boracic lint dressings. (*g*) Wasting. Massage and passive movements will promote tissue nutrition. Iron and hæmoglobin should be given with the food which should consist chiefly of milk and eggs, while tropon or plasmon may be given instead of meat. The remarks made above on beef-tea and meat extracts also apply here. (*h*) Suicidal tendencies. These should be strictly guarded against by careful supervision. Sharp instruments, or objects wherewith wounds might be self-inflicted, should not be left within the patient's reach, and strings and ligatures (which might be used for tying round the throat with suicidal intent) must not be allowed to remain in the patient's possession either during the day or at bed-time. A male patient who is suffering from melancholia, however mild, should be advised to grow a beard, so that shaving will be unnecessary, and the temptation of having a razor in hand is obviated. (*k*) Exercise. When the acute stage of melancholia has subsided, when the bowels and digestion are resuming their normal condition, and insomnia has been overcome, the patient may be exercised daily in walking, visits, or amusements generally, to a moderate degree, so as not to produce exhaustion or marked fatigue, and this may be continued till strength is gradually acquired.

During this period baths, in the form of shower and spinal douches, electric baths, and the application of the constant current to the spine and scalp (Benedikt), will promote recovery. The patient's daily routine of life should be well ordered and regulated till recovery is completed and he is enabled to resume his occupation or business in life. The visits of friends and relatives may be encouraged during the stage of convalescence. (*l*) General remarks. Where there is much stupor or inertia in a patient who has been acutely melancholic for a brief period special attention should be paid to the measures recommended above under headings (*a*) (*d*) (*g*) (*k*). And in all cases it should be borne in mind that, while the various symptoms recorded above seriatim in regard to treatment are not neglected, the primary therapeutic indication is to treat the brain as directly as possible. Hence the necessity for rest from work and worry, sleep, the regulation of diet, and the administration of such drugs as strychnine, iron and hæmoglobin, mercurials, the hypophosphites, opium, sulphonal and paraldelyde, and others which, while acting as nerve-tonics and sedatives also promote elimination from the bowel and bladder, and tend to relax the high arterial tension—in a word rest and sleep, suitable dieting, aperients to promote elimination and diminish toxæmia from the alimentary canal, and nerve-tonics (with hypnotic measures where necessary) sum up the principles of treatment. In regulating and ordering treatment generally regard must be had to the patient's age and condition as regards marriage, as the fact of being married or single, of being pregnant or in the puerperal state, of being an adolescent, or at the climacteric period of life, will necessitate special lines of treatment being followed. Moreover, in cases of melancholia from stresses of exceptional severity such as seduction and desertion in the young, or sudden ruin and the loss of the fruits of a life's work in middle-aged or elderly persons, treatment will have to be modified partly according to the value and potency of these ætiological factors. In obstinate cases of refusal of food, forced feeding will have to be adopted. The patient should be fed through an indiarubber œsophageal tube (passed a few inches into the œsophagus and with a funnel attached to the upper end) with a custard composed of milk and eggs (one egg to one pint of milk), flavoured with a tablespoonful of port wine or brandy, and to which a little tropon or plasmon has been added. Much of the treatment above recommended can be effectively carried out only at an asylum or special institution for mental diseases. Even mild cases of melancholia are, as a rule, not suited for travelling, and the danger of suicide is ever present under such circumstances. Coca wine may be taken by a patient twice a day, a wineglassful at meals, in mild melancholia, such as often follows influenza. The use of drugs such as cocaine or morphia hypodermically in treating acute symptoms (including insomnia) must be left in the hands of the physician alone, and not relegated to others. As soon as convalescence has begun physical occupation should be attempted. Walks and drives, field-studies in natural history (botany, geology, etc.), gardening and agriculture, golf, bicycling, lawn-tennis, croquet, bowls, and other light out-door and open-air pursuits should be encouraged. (Fishing or boating, perhaps, are better postponed till recovery is complete, and all danger of suicidal impulses has passed away.) Travelling and "distraction," at one time supposed to be a proper method of treatment for melancholia, must not be advised in the early stages of the disease. "Distraction by means of travels, entertainments, etc., is to be avoided by all means, and rest is the only proper treatment at the outset."[3] All excitement, emotional sexual and religious, must be avoided during the course of the disease. "During convalescence, but only at that period, the patient may receive the comforts of religion."[4]

[1] Haig, Uric Acid and Disease (1900). [2] Among the chief authorities who recommend and adopt this are Krafft-Elring (Lehrbuch der Psychiatrie), Schüle, Griesinger, Magnan, Batty-Tuke (discussion at the British Medical Association at Bristol, 1894, Psychology Section), Crichton-Browne, Spitzka (Text-book of Insanity), Petersen (Text-book of Mental Diseases), Morselli, and Korsakoff (Cours de Psychiatrie). [3] Kirchoff, Handbook of Insanity (1893). [4] Ibid.

